# The factor structure of the 12-item general health questionnaire (GHQ-12) in young Chinese civil servants

**DOI:** 10.1186/s12955-016-0539-y

**Published:** 2016-09-26

**Authors:** Ying Liang, Lei Wang, Xican Yin

**Affiliations:** 1 Department of Social Work and Social Policy, School of Social and Behavioral Sciences, Nanjing University, Nanjing, 210023 Jiangsu Province People’s Republic of China; 2 College of Sciences, Northeastern University, Shenyang, People’s Republic of China

**Keywords:** Young Chinese civil servant, GHQ-12, Factor structure, Scale, Quantitative methods

## Abstract

**Background:**

The 12-item General Health Questionnaire (GHQ-12) is a commonly used screening instrument for measuring mental disorders. However, few studies have measured the mental health of Chinese professionals or explored the factor structure of the GHQ-12 through investigations of young Chinese civil servants.

**Method:**

This study analyses the factor structure of the GHQ-12 on young Chinese civil servants. Respondents include 1051 participants from six cities in eastern China. Exploratory Factor Analysis (EFA) is used to identify the potential factor structure of the GHQ-12. Confirmatory Factor Analysis (CFA) models of previous studies are referred to for model fitting.

**Results:**

The results indicate the GHQ-12 has very good reliability and validity. All ten CFA models are well fitted with the actual data.

**Conclusion:**

All the ten models are feasible and fit the data equally well. The Chinese version of the GHQ-12 is suitable for professional groups and can serve as a screening tool to detect anxiety and psychiatric disorders.

## Background

The 12-item General Health Questionnaire (GHQ-12) originated from a general health questionnaire designed by Goldberg, which reflects the mental health condition of respondents by 12-item self-assessment results. It has been widely applied to clinical patient’ psychological assessment and self-assessment of the general population [1]. Little research has been initiated to survey Chinese occupational groups by the GHQ-12; meanwhile, no researchers have detected the factor structure of the GHQ-12 via investigations of the high anxiety-risk occupational groups, including those young civil servants working at grassroots government agencies. In this paper, the GHQ-12 is used in the measurement of young Chinese civil servants’ mental health to analyse the factor structure of GHQ-12 in this occupational group.

### Application of GHQ-12

General Health Questionnaire (GHQ), developed by British scholar Goldberg in 1972, is one of the most popular and widely used screening instruments for recognition and measurement of mental health [2]. The original GHQ consists of 60 items, and now there are multiple versions, including GHQ-1, GHQ-12, GHQ-20, GHQ-28 and GHQ-30. Among these, the GHQ-12 is the most popular due to its simplicity [2, 3]. The GHQ-12 consists of 12 items, each of which is evaluated by four indexes. The two most commonly used scoring types are the bi-modal (0-0-1-1) and Likert scoring methods (0-1-2-3) [4].

The GHQ-12 has satisfactory reliability [5–7] and good sensitivity and specificity [8, 9]. It has been applied to different populations in different countries to study its reliability and validity, and to explore the mental disorders characteristics of the populations in previous studies. These respondents have included both adolescents [4, 7, 10] and adult community residents [1, 11]. Numerous studies have focused on occupational groups, including nurses [12], academic staff [13], cohort [11] and civil servants [14] etc. Simultaneously, different language versions of the GHQ-12 have proven acceptable, (e.g., Arabic [9], Australian English [13], German [2], Spanish [10] and Swedish [15]). Although few studies have focused on the applicability of the Chinese version of the GHQ-12, it has been proven that the Chinese version is reliable and valid [16, 17]. Scholars have applied the Chinese version of the GHQ-12 to university students [16], and the results have shown that the instrument is acceptable. It has also been used to evaluate the mental disorders of secondary school students, reaching the same conclusion [17]. The studies mentioned above were focused on students, but there is scarce literature on the applicability of the GHQ-12 to specific Chinese occupational groups. Although previous studies have used the GHQ-12 to measure the mental health of civil servants and nurses, no research has been done on young civil servants in grassroots government agencies, a group that has specific occupational and physical characteristics.

### Mental disorders of civil servants

Various populations in China suffered from mental disorders [18–21]. Among these, civil servants are professionals with a high risk of mental disorders [22]. Civil servants are responsible for managing the state, and they link average citizens and the government [23]. The nature of their work and the organisational environment determines the specificity of their work, and their occupational health has specific characteristics. In fact, previous studies have shown that the health condition of this occupational group is not optimistic, and even can be worrisome [24].

Civil servants are under high work-related pressure, resulting in poor mental and physical health and lack of support [25, 26]. In addition, their high-intensity work makes them more vulnerable to disease. Most young civil servants who have recently entered the executive authorities are in lower-level positions. Marmot [27] conducted a study finding that mortality rate due to coronary heart disease of male civil servants in low-level positions was three to six times higher than that of male civil servants in higher positions in London. Civil servants in low-level positions have been employed for a short time [28], have significant employment pressure [29], tend not to play sports and are more susceptible to high blood pressure and blood sugar [30]. In addition, negative health behaviours and habits among civil servants are quite common, including addicted to tobacco and alcohol, lack of exercise and a tendency toward chronic diseases like insomnia [31–34]. Thus, concerns about the health, especially the mental disorders, of special occupational groups are of great importance and urgency.

### Existing factor structures of the GHQ-12

At present, the existing researches on the factor structure of the GHQ-12 have been full of controversy, as the results have been inconclusive. The majority of previous studies extracted one to three factors from 12 items. Initially, the GHQ-12 was designed as a unidimensional scale that all projects included in a main factor, but only a few scholars supported this original one-factor model in subsequent empirical studies. Banks [5] and his colleagues conducted three separate investigations, to test the validity for three types of samples: employees of an engineering firm, recent leavers and unemployed men. The results showed that the scaling properties of the GHQ-12 are sufficiently good to justify the use of a single-scale score. However,the original unidimensional model may be unable to provide researchers sufficient information because of its simplicity [5]. Since then,a number of studies have explored its possible factor structures and revealed different dimensions in the structure of the GHQ-12, mainly with two or three factors. A survey of teachers in Western Australia found that the GHQ-12 contains two dimensions, and they were described separately in a statement by positive and negative words [35]. This result also repeatedly showed the adequate fit to the data [15, 36, 37]. In another study, using a large sample of young Australians (*n* = 8998), Graetz [10] extracted three factors from the GHQ-12: Social Dysfunction (including items 1, 3, 4, 7, 8 and 12), Anxiety and Depression (including items 2, 5, 6 and 9), Loss of Confidence (including items 10 and 11). With the application of the confirmatory factor analysis, many studies have suggested that this three-factor model, compared to other models, has a better goodness of fit [7, 38, 39]. Simultaneously, other scholars have also found that the GHQ-12 has three dimensions [9, 40],but named the factors differently from Graetz [10]. However, the validity and usefulness of these multi-dimensional solutions have frequently been questioned because of the high degree of correlation between the factors [7, 41]. Other studies have focused on the connections between wording effect and negative vocabulary. Hankins [12] found through statistics from the Health Survey for England that the unidimensional model, with correlated errors on the negatively worded items, was superior to the two-factor (positively and negatively worded items) or three-factor models proposed [42]. The study results of Li [17] and Aguado are consistent with this. Both contend that as long as the wording effect is controlled, the GHQ-12 is a unidimensional model [17, 43]. In general, although there have been many studies on the factor structure of the GHQ-12, they have not reached a unanimous conclusion. Thus exploration and verification of the factor structure of the GHQ-12 are still necessary.

### Research objectives

As it is a constantly improved and widely used measurement tool for mental health, the GHQ-12 has been applied to different populations in many countries. The research subjects have mostly been members of the general population and certain occupational groups. No paper that has made its sample young civil servants working at grassroots government agencies. What is more, the Chinese version of the GHQ-12 has not been researched extensively; few studies have examined the factor structure and psychological characteristics of this scale in Chinese occupational groups. In this paper, we use the GHQ-12 to measure the mental health of young Chinese civil servants and to analyse the GHQ-12 factor structure in this occupational population. This study has the following three aims: (1) Creatively applying the GHQ-12 to young civil servants working in grassroots government agencies to broaden the scope of use and acceptance of the GHQ-12; (2) Researching the GHQ-12 factor structure of occupational groups in China to provide more extensive clinical and empirical data for the improvement and development of the GHQ-12; (3) Through studying the reliability and validity of the GHQ-12 to promote its use in Chinese clinical measurements and individual self-assessment.

## Methods

### Samples

The subjects of this study are young civil servants working at 24 grassroots government agencies. We designed and conducted a cross-sectional study and selected six cities with comparatively developed economy in eastern China (Nanjing, Shanghai, Suzhou, Hangzhou, Yangzhou, Wenzhou). These cities are under better urban construction, and they have more complete and diverse government agencies, so they make representative sample regions. We randomly selected civil servants under 45 years old working in administrative departments below the county level. In the process of distributing the questionnaire, we asked whether respondents were willing to accept the investigation and state principles, such as the confidentiality and authenticity, in advance. Respondents were asked to complete the questionnaire independently. If they did not understand any item, they could consult the investigator on the spot. Finally, 1200 questionnaires were distributed, (i.e., 200 for each city). A total of 1051 questionnaires were returned, making the response rate 87.58 %.

### Instruments

This study used the GHQ-12 proposed by Goldberg [44] to measure the mental health of young Chinese civil servants. This questionnaire includes 12 items (six positively worded items (e.g., Have you been able to concentrate on whatever you are doing?) and negatively worded items (e.g., Have you lost much sleep over worry?)). We adopted the four-point Likert scale, with each item ranging from 0 to 3. For negatively worded items, ‘0’ indicated Not at all, ‘1’ indicated Seldom, ‘2’ indicated Usual and ‘3’ indicated More than usual, while positively worded items were reversely scored. All items were added to obtain the total score, making the score range 0-36 (with a higher score indicating worse mental health). Scores over the cut-off point of 12 could be classified as cases [8].

### Statistical analysis

First, the score distributions for each item and the whole scale were calculated to understand the mental health of young Chinese civil servants. Then, we conducted a reliability analysis of the GHQ-12 scale. Finally, we used factor analysis to explore and validate the GHQ-12 factor structure of the respondents. In the factor analysis process, the total sample (*n* = 1051) was randomly split into an Exploratory Factor Analysis (EFA) sample (*n* = 525) and a Confirmatory Factor Analysis (CFA) sample (*n* = 526) by SPSS 19.0. Then, EFA was conducted to explore the underlying factor structure of the GHQ-12 in the EFA sample using the principal axis extraction method with varimax rotations. Parallel analysis was the method used to decide the number of factors extracted from EFA [45]. CFA with maximum likelihood estimation (MLR) was referred to for model fit.

The selection of the models for CFA was based on a literature review. We referred to a large number of previous studies and selected the model with the best fit from each validation study. As mentioned in the introduction, most of the studies extracted one to three factors from 12 items. The models most strongly supported the unidimensional model with wording effect by Hankins [41], Andrich and Van Schaubroecks’ two-dimensional model [35] and the three-dimensional model by Graetz [10], which have been supported and validated by many other scholars. Furthermore, an additional five two-dimensional and three-dimensional models [6, 9, 40, 46, 47] were also included, which have been proven to fit well by previous studies. Finally, coupling the unidimensional model from the original GHQ-12 designed by Goldberg and our model results of EFA, CFA was then performed by testing ten competing factor models with one-, two- and three-dimensional solutions of the CFA sample. The ten types of measurement models were as follows:(1) unidimensional model (original), (2) unidimensional model with correlated errors on the negatively worded items by Hankins [41], (3) two-dimensional model proposed by Andrich and Van Schaubroeck [35] (positively worded items (including items 1, 3, 4, 7, 8 and 12) and negatively worded items (including items 2, 5, 6, 9, 10 and 11)), (4) two-dimensional model by Schmitz et al. [46] (Anxiety/Depression (including items 1, 2, 6, 7, 10 and 11), Social Performance (including items 4, 5, 8, 9 and 12)), (5) two-dimensional model by Politi et al. [6] (Dysphoria (including items 2, 5, 6, 9, 10 and 11) and Social Dysfunction (including items 1, 3, 4, 7 and 8), (6) three-dimensional model by Graetz [10] (Social Dysfunction (including items 1, 3, 4, 7, 8 and 12), Anxiety and Depression (including items 2, 5, 6 and 9), Loss of Confidence (including items 10 and 11), (7) three-dimensional model by Farrell [47] (Anxiety (including items 2, 5, 10, 11 and 12), Depression (including items 1, 6, 7, 8 and 9) and Social Dysfunction (including items 3 and 4), (8) three-dimensional model by Daradkeh et al. [9] (General Dysphoria (including items 5, 6, 9, 10 and 11), Lack of Enjoyment (including items 1, 2, 7,8 and 12) and Social Dysfunction (including items 3 and 4), (9) three-dimensional model by Martin [40] (Cope (including items 1, 3, 4, 8 and 11), Stress (including item 2, 5 and 7) and Depression (item 6, 9 and 12), (10) unidimensional model obtained from EFA.

The reasons we conducted EFA before CFA are as follows: First, previous studies on the factor structure of the GHQ-12 have been full of controversy, and they have been inconclusive. The names of each factor in the same dimensions model also vary in the existing factor dimensional model. Second, so far, no studies have conducted EFA on Chinese occupational groups, although Ye [16] and Li [17] have applied EFA to Chinese students. Last, to the best of our knowledge, this is the first study of the GHQ-12 factor structure on young Chinese civil servants. Young civil servants are a specific occupational group at a high risk of mental disorders. They bare a greater work pressure and higher propensity for mental disorders [25, 26]. The results of the general population or other occupational groups may not be applicable to young civil servants.

We used EPIDATA3.1 double entry to test the sample quality. The significance level for all tests was 0.05. We performed the CFA model with AMOS 21.0 and EFA models and other statistical tests with SPSS 19.0. Thus, we adopted this short analysis to explore the reliability and validity of the GHQ-12 on young Chinese civil servants.

## Results

### Demographics

The demographic and socioeconomic characteristics of respondents mainly included gender, age, education, employment level and marital status. Of the 1051 respondents, 61.5 % were male, and 38.5 % were female. In terms of age, 41.5 % of respondents were aged 20–29 years, 48.4 % of respondents were aged 30–39 and 10.1 % were aged 40–45. Education levels of these young civil servants were divided into four categories: Junior college and bellow, Undergraduate, Graduate and Doctorate. Most respondents fell into the Junior college and below (32.7 %) and Undergraduate (42.6 %) categories, and most respondents were staff and below (41.4 %), and not married (46.8 %). In addition, the EFA sample and the CFA sample had similar distributions of age, education and employment grade. The CFA sample had a high ratio of respondents who were male or married. In general, the demographics of the respondents are representative of the young Chinese civil servants. Table 1 provides descriptive statistics of the sociodemographic characteristics of the respondents in this study.

**Table 1:** Sociodemographic characteristics of participants

Demographics	Total sample (*n* = 1051)		EFA sample (*n* = 525)		CFA sample (*n* = 526)	
	N	%	N	%	N	%
Gender						
Male	640	61.5	309	59.3	331	63.7
Female	401	38.5	212	40.7	189	36.3
Age (years)						
20–29	434	41.5	227	43.5	207	39.5
30–39	506	48.4	250	47.9	256	48.9
40–45	106	10.1	45	8.6	61	11.6
Education						
Junior college and bellow	341	32.7	173	33.3	168	32.1
Undergraduate	445	42.6	233	44.8	212	40.5
Graduate	204	19.5	92	17.7	112	21.4
Doctor	54	5.2	22	4.2	32	6.1
Employment Level						
Staff and below	434	41.4	228	43.6	206	39.2
Township	327	31.2	163	31.2	164	31.2
County deputy	175	16.7	84	16.1	91	17.3
County chief	112	10.7	48	9.2	64	12.2
Marital status						
Unmarried	486	46.8	252	48.7	234	44.9
Married	373	35.9	172	33.3	201	38.6
Divorced or others	179	17.2	93	18.0	86	16.5

### Descriptive and health characteristics

Table 2 shows the overall and individual item scores of the GHQ-12. The GHQ-12 items were to the left on both sides of the score distribution except item 10, and the kurtosis coefficients were less than 0. The average score of the GHQ-12 was 23.62 (SD = 7.92), which was far higher than the cut-off point of 12 [8], and of all the respondents, 86.49 % scored greater than or equal to 12 and could be used as cases. This indicated that the health, especially the mental health, of the respondents was in very poor condition. In particular, the highest average scores were for items 1, 9 and 11, which were more than 2.10. Of these items, the average score of item 9 was 2.14 (SD = 1.08), as the highest, indicating that the majority of respondents felt unhappy and depressed. While the average score of item 12 was 2.06 (SD = 1.06), the majority of respondents (74.3 %) scored 2 or 3 points, and only 13.8 % of respondents scored 0, showing that in general the respondents did not feel happy.

**Table 2:** Descriptive statistics for GHQ-12 items and summary scores (*N* = 1051)

GHQ-12 items	Mean	SD^a^	Skewness	Kurtosis	Response frequencies (%)			
					0	1	2	3
1. Able to concentrate	2.10	1.05	−0.893	−0.484	13.6	10.3	29.0	47.1
2. Lost much sleep	1.99	1.05	−0.676	−0.791	13.2	15.6	29.9	41.3
3. Playing a useful part	1.94	1.13	−0.601	−1.084	17.2	15.2	23.9	43.7
4. Capable of making decisions	2.01	1.10	−0.709	−0.882	15.1	14.2	25.4	45.3
5. Under stress	1.85	1.06	−0.455	−1.032	14.7	20.1	30.5	34.7
6. Could not overcome difficulties	1.90	1.13	−0.548	−1.133	18.0	15.5	25.2	41.3
7. Enjoy your day-to-day activities	1.99	1.09	−0.658	−0.944	14.9	15.7	25.2	44.1
8. Face up to problems	2.02	1.12	−0.785	−0.816	17.3	9.6	26.6	46.4
9. Feeling unhappy and depressed	2.14	1.08	−0.939	−0.505	13.7	10.8	23.6	52.0
10. Losing confidence	1.50	1.17	0.008	−1.479	27.9	22.5	21.3	28.3
11. Thinking of self as worthless	2.12	1.08	−0.869	−0.662	13.3	13.4	21.2	52.0
12. Feeling reasonably happy	2.06	1.06	−0.813	−0.620	13.8	11.9	29.2	45.1
Mean GHQ-12 score	23.62	7.92						

^a^ *SD* standard deviation

A higher score indicates a worse situation

### Reliability and correlations analysis of GHQ-12

Cronbach’s alpha reliability was obtained using the Spearman-Brown formula. Table 3 shows the reliability and correlations between items and for the overall GHQ-12 scale. Cronbach’s alpha of the GHQ-12 was 0.844 (>0.8), indicating that the scale has a good reliability and correlations. Item 11 had the highest correlation coefficient, which was 0.609. Correlation coefficients for the rest of the items and the total score ranged from 0.324 to 0.606. The Cronbach’s alpha coefficients when an item was deleted were over 0.8, but below the total coefficients, they range from 0.824 to 0.837 when an item was deleted, indicating that each item was necessary and of equal importance.

**Table 3:** Correlations between items and for overall GHQ-12 scale

GHQ-12 items	Correlation of item with overall scale	Cronbach’s alpha if the item is eliminated
1. Able to concentrate	0.519	0.831
2. Lost much sleep	0.324	0.845
3. Playing a useful part	0.530	0.830
4. Capable of making decisions	0.541	0.829
5. Under stress	0.480	0.834
6. Could not overcome difficulties	0.454	0.836
7. Enjoy your day-to-day activities	0.478	0.834
8. Face up to problems	0.589	0.826
9. Feeling unhappy and depressed	0.537	0.830
10. Losing confidence	0.440	0.837
11. Thinking of self as worthless	0.609	0.824
12. Feeling reasonably happy	0.606	0.825
Cronbach’s alpha	0.844	

### Factor analysis

Previous studies on the factor structure of the GHQ-12 have been full of controversy, and they have been inconclusive. In the factor analysis process, the total sample (*n* = 1051) was randomly split into the EFA sample (*n* = 525) and the CFA sample (*n* = 526) by SPSS 19.0. Using the data from the EFA sample, EFA was firstly used to identify the possible latent variables of the GHQ-12. Table 4 shows the results of the EFA of the scale using the principal axis extraction method with varimax rotation. Kaiser-Meyer-Olkin (KMO) measured the sampling adequacy of the GHQ-12 as 0.908 (>0.9), and the Bartlett test results passed the significance test (approximated chi-square = 1249.186, df = 66, *P* < 0.001), indicating the adequacy of the sample. We extracted three factors: Factor 1, Factor 2 and Factor3. The eigenvalue of each factor was greater than 1, and the load factor was greater than 0.4 for each item. Factor 1 included six items, items 4, 6, 9, 10, 11 and 12, and explained 21.206 % of the variance. Factor 2 included four items, item 3, 5, 7 and 8, and explained 17.339 % of the variance. Factor 3 included two items, items 1 and 2, and explained 11.675 % of the variance. The three factors together explained 50.220 % of the total variance.

**Table 4:** EFA for GHQ-12 (*n* = 525)

GHQ-12 items	Factor loadings		
	Factor 1	Factor 2	Factor 3
1. Able to concentrate			0.555
2. Lost much sleep			0.855
3. Playing a useful part		0.660	
4. Capable of making decisions	0.586		
5. Under stress		0.479	
6. Could not overcome difficulties	0.628		
7. Enjoy your day-to-day activities		0.789	
8. Face up to problems		0.509	
9. Feeling unhappy and depressed	0.554		
10. Losing confidence	0.692		
11. Thinking of self as worthless	0.606		
12. Feeling reasonably happy	0.489		
% of variance	21.206	17.339	11.675
Cumulative % of variance	21.206	38.545	50.220
KMO	0.908		
Bartlett’s test of sphericity	Chi^2^ = 1249.186	df = 66	Sig. = .000

In reference to the historical study in which the GHQ-12 measured different populations, we obtained the CFA models. Having obtained the factor structure of our EFA, we used the CFA sample (*n* = 526) to perform CFA on the ten confirmatory models.

Table 5 shows the goodness of fit indexes for the CFA model. The statistics results indicate that all the models were well fitted with the actual data. In CFA, the fit indices of all models were relatively similar, and RMSEA was less than 0.05 (approximately 0.03). CFI and TLI were also greater than 0.9, and the chi-square/degrees of freedom ratio was less than 2, ranging from 1.465 to 1.561. This indicated that all of the ten confirmatory models for the GHQ-12 did equally well and had satisfactory levels of fit.

**Table 5:** Goodness of fit indexes for CFA model (*n* = 526)

Models	χ^2^	df	χ^2^/df	CFI	TLI	RMSEA
Unidimensional model (Original)	79.370	54	1.470	0.985	0.978	0.030
Unidimensional model with correlated errors (Hankins)	57.154	39	1.465	0.989	0.979	0.030
Two-dimensional model (Andrich and Van Schoubroeck)	79.104	53	1.493	0.985	0.977	0.031
Two-dimensional model (Schmitz et al.)	65.515	43	1.523	0.985	0.976	0.032
Two-dimensional model (Politi et al.)	71.938	43	1.673	0.980	0.975	0.036
Three-dimensional model (Graetz)	76.732	51	1.505	0.985	0.977	0.031
Three-dimensional model (Farrell)	63.993	41	1.561	0.985	0.976	0.033
Three-dimensional model (Daradkeh et al.)	79.007	51	1.549	0.983	0.975	0.032
Three-dimensional model (Martin)	60.887	41	1.485	0.987	0.977	0.031
Three-dimensional model (EFA)	76.112	51	1.492	0.985	0.977	0.031

## Discussion

This study examined young civil servants working at grassroots governmental agencies in six Chinese eastern developed cities, used the Chinese version of the GHQ-12 to measure their psychological characteristics of mental health and analysed the reliability and validity of the scale. Although many previous studies have reported the reliability and validity of the GHQ-12 on the Chinese population [16, 17, 48], to the best of our knowledge, this is the first study of the GHQ-12 factor structure on young Chinese civil servants working in grassroots government agencies. The GHQ-12 surveyed young civil servants belonged to an occupational group at a high risk of mental illness, breaking the previous trend in the literature of focusing only on other occupational groups or the general population. We studied the factor structure of the Chinese version of the GHQ-12 to provide more empirical data for the development of the GHQ-12 and the progress of mental health measurement.

For the reliability, the Cronbach’s alpha value of 0.844 indicated the satisfactory reliability and internal consistency of the GHQ-12 for young Chinese civil servants in eastern China, which was similar with the values reported in other populations [6, 7, 17]. Simultaneously, the coefficient values excluding Cronbach’s alpha of individual items were lower than the total coefficient value, and they were all greater than 0.8, ranging from 0.824 to 0.837. This proves that taking out any item may decrease the credibility of the total scale. Thus for measuring the mental health status of young Chinese civil servants, each item of the GHQ-12 scale is of equal importance.

The EFA extracted three factors (Factor 1, Factor 2, Factor 3) from the GHQ-12 in our study, and these three factors can explain 50.220 % of the overall variance. Factor loadings were also high (0.479–0.855). Factor 1 included items 4,6,9,10,11 and 12, Factor 2 included items 3,5,7 and 8 and Factor 3 included items 1 and 2. In this article, the results of exploring the GHQ-12 factor structure were similar to those of many other international versions [9, 10, 39]. That is that the three factors structure is more suitable for the GHQ-12. By studying a large sample of young Australians, Graetz [10] pointed out that the structure of the GHQ-12 included three separate dimensions: Anxiety/Depression, Low Social Function and Deficiency of Confidence. This result was verified and supported by many other studies [10]. Some scholars, such as Daradkeh [9] and Martin [39], also found that the GHQ-12 was three-dimensional, but they used different names from those used by Graetz [10].

Finally, the present study explored the factor structure of the GHQ-12 using a CFA approach with ten different dimensional models. One model was the original unidimensional structure. Eight models were based on the literature providing details of the CFA of the GHQ-12. And the remaining one was obtained from the EFA. The results of the CFA showed that the goodness-of-fit indices of the ten models are similar and all of these models are feasible and fit the data equally well. We cannot accurately identify which model is the best one underlying the data. This outcome seems to be rarely consistent with the previous studies, and only one paper to the date obtained the same conclusion [38]. As noted by Campbell et al., a number of different models examined in their studies had satisfactory fit to the Tasmanian data when the original scoring was used, indicating that CFA cannot identify the true model [38]. Furthermore, the fitness of the EFA model is not the best in our research. Of course, this is not an uncommon occurrence; rather, we can see it in previous studies [7, 17, 41]. It is mainly because when we performed EFA, the number of factors extracted was decided by arbitrary criteria, without consideration of the theory-driven frame of the model dimensions, while the CFA was theory-driven, making the verification results of the two different [49]. Besides, the CFA used more fitness indexes than the EFA to study and evaluate the factor structure of the scale, making the result more accurate [50]. In addition, it is usually assumed that one item is loaded in one factor in CFA, yet every factor finally relies on all the common factors in EFA, making it relatively difficult to explain the results [7].

Nevertheless, our study has certain limitations. First, the sample data were selected from six of the most developed cities in eastern China: Nanjing, Shanghai, Suzhou, Hangzhou, Yangzhou and Wenzhou. We did not include some undeveloped cities in the northern and western areas, meaning the range was relatively small. Moreover, our sampling strategy (i.e., using 200 individuals from each of the six cities, without using sampling weights to account for the differences in the populations among these cities), probably did not result in a random sample of this population and might have skewed the results. Furthermore, young civil servants are a specific occupational group at a high risk of mental disorders, facing specific professional circumstances and work environments. Therefore, the results may not be applicable to the general population or other occupational groups. In addition, the results of the CFA showed that all ten models fitted the data equally well. Thus we cannot accurately identify and determine the precise factor structure of the GHQ-12 and can only provide an initial exploration for the factor structure and its development. More studies will be needed in order to achieve an advanced analysis and verification of the reliability and validity of the GHQ-12.

## Conclusion

The 12-item General Health Questionnaire (GHQ-12) has been widely used as a measure of minor psychiatric disorders, but there has been no study on this occupational group of young civil servants in the grassroots government agencies. This study examined samples of young civil servants from 24 grassroots government agencies in China to analyse the factor structure of the Chinese version of the GHQ-12 on this group. The results indicated that the respondents had higher scores than average, meaning they were in a poorer mental health condition. The GHQ-12 has a high reliability and validity in the young Chinese civil servants and the clinical and psychometric performance of the scale was good. Our study found that all the ten models were feasible and fitted the data equally well. Overall, the Chinese version of the GHQ-12 is suitable for professional groups and can serve as a screening tool to detect anxiety and psychiatric disorders.
